# Supplementary material for: 3D ink-extrusion additive manufacturing of CoCrFeNi high-entropy alloy micro-lattices
Source: Nat Commun. 2019 Feb 22;10:904. doi: 10.1038/s41467-019-08763-4 (PMC6385271; doi:10.1038/s41467-019-08763-4)
Supplement: Supplementary file 1 — Supplementary Information [file 41467_2019_8763_MOESM1_ESM.pdf]

Supporting Information

3D ink-extrusion additive manufacturing of CoCrFeNi high-entropy alloy micro-lattices

Kenel et al.

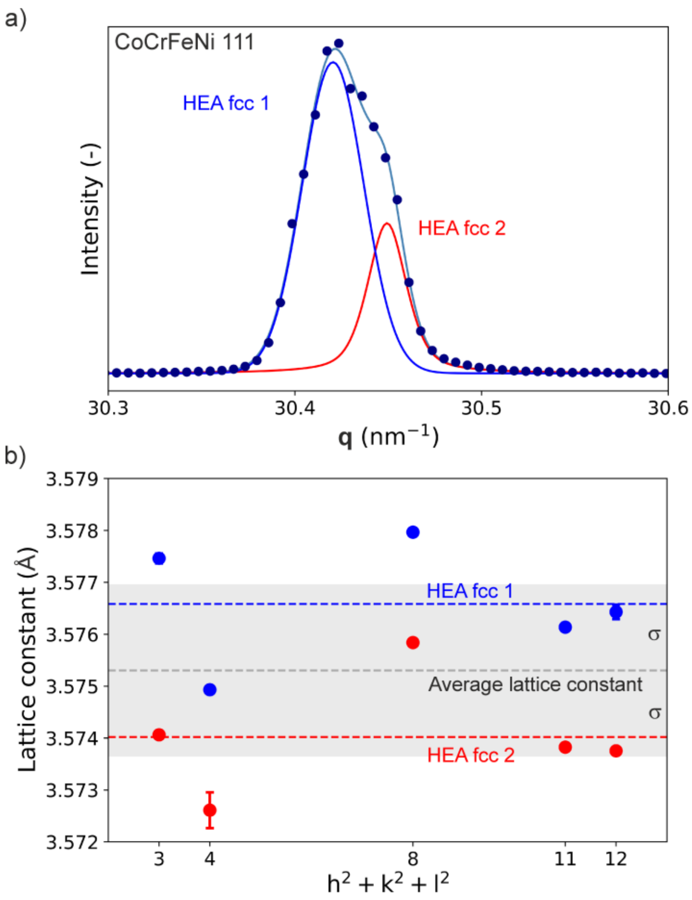

Supplementary Figure 1 Evidence of two coexisting face-centered cubic phases in co-reduced CoCrFeNi HEA filaments. a) Detail of the skewed CoCrFeNi fcc 111 diffraction peak, measured at room temperature, with its fitted deconvolution into two symmetric Pseudo-Voigt profiles (blue and red line) that reproduce the measured peak profile. b) Calculated lattice constants of the two fcc phases using the 111, 200, 220, 311 and 222 peaks fitted with two Pseudo-Voigt profiles. Average values for both fcc phases and a global average are indicated. Error bars correspond to ±σ.

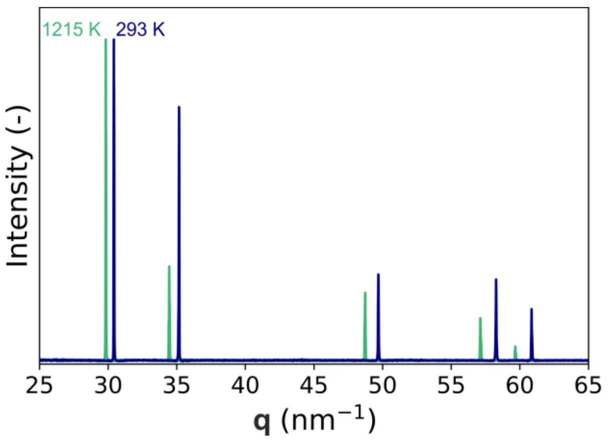

Supplementary Figure 2 Thermal expansion of the fcc HEA lattice. Normalized diffractograms acquired at 1215 K (green) and after cooling to room temperature (blue) showing the thermal expansion of the fcc HEA lattice. Changes in relative peak height indicate texture effects due to limited grain statistics in the sintered filament. The lattice parameters are used to calculate an average coefficient of thermal expansion.

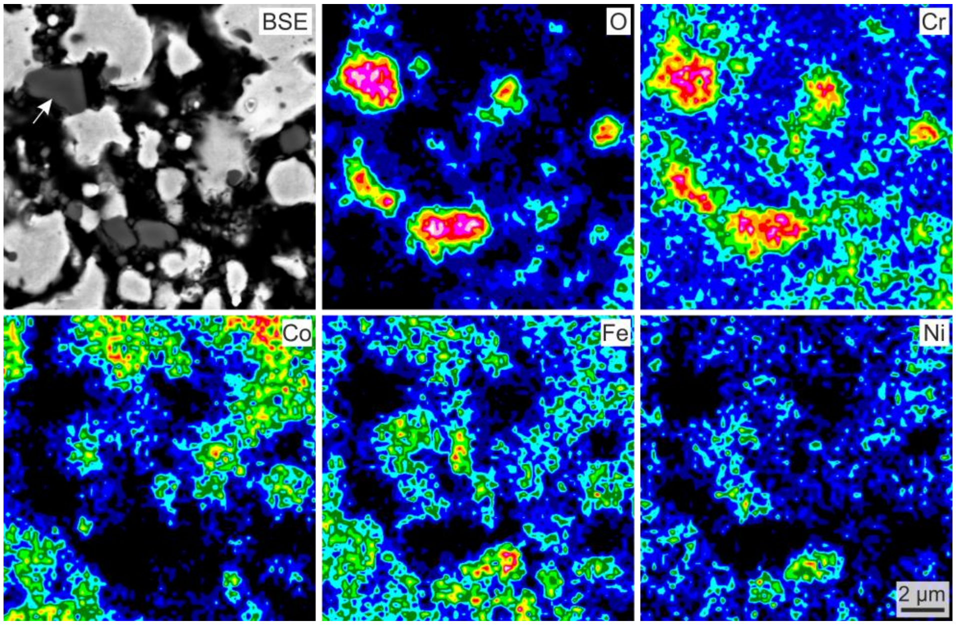

Supplementary Figure 3 Elemental distribution in a partially co-reduced CoCrFeNi HEA filament. SEM-EDS mapping of the center filament in Figure 3 sintered at 1173 K for 1h. The elements O (top, center) and Cr (top, right) are clearly co-located, showing the presence of Cr_2O_3, visible as grey particles in BSE (top, left, arrow). Co, Fe and Ni, together with reduced Cr, are forming the surrounding metallic matrix (bottom). Full interdiffusion and homogenization has not yet been achieved thus creating compositional variation.

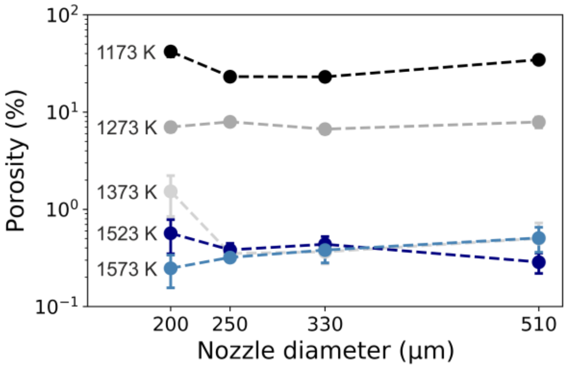

Supplementary Figure 4 Process stability with different extrusion nozzles. Influence of extrusion nozzle diameter on the observed porosity of HEA filaments, after sintering at various temperatures between 1173 and 1573 K for 1h. Independently of the nozzle and resulting filament diameter, very low porosities (0.2-0.5%) are achieved after sintering at 1573 K. Error bars correspond to $\pm\sigma$.

Supplementary Table 1 Summary of reported mechanical properties and grain size for equiatomic CoCrFeNi at room temperature

Method / Geometry	Specimen diameter (mm)	Yield stress (MPa)	Ultimate tensile strength (MPa)	Elongation to failure (%)	Grain size (µm)	Reference
Cold-drawn wire	7	1107	1107	12.6	4.1	Huo et al.[1]
Cold drawn + recrystallized wire	7	288	NA	NA	NA	
Arc melted, dog-bone	1x0.5	200	370	41	200-300	Huo et al.[2]
Arc melted, dog-bone	6	229	454	36	NA	Huang et al.[3]
Printed, reduced from oxides, sintered filament	0.125	250±5	598±8	33.8±1.3	35-80	This work

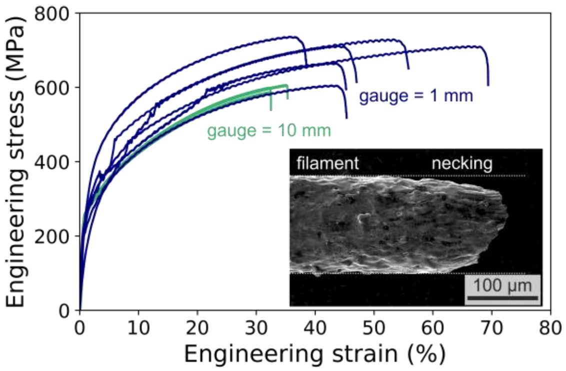

Supplementary Figure 5 Evidence for oligo-crystalline tensile behavior of short HEA filaments. Stress-strain curves of sintered CoCrFeNi HEA filaments with a gauge length of 1 mm (blue) and 10 mm (green) and an average diameter of 125 µm. Tensile curves for individual short filaments (1 mm gauge) do not overlap, and also show serrations and jumps, indicating that slip systems in single grains in favorable orientations are activated and then strain-hardened. Testing of short filament segments (n=6) yields higher ultimate tensile strength and ductility compared to 10 times longer filaments (10 mm gauge, n=3) with necking clearly observed in all specimens. Inset: Micrograph of a fractured filament (1 mm gauge) showing formation of a neck within 100 µm of the fracture zone.

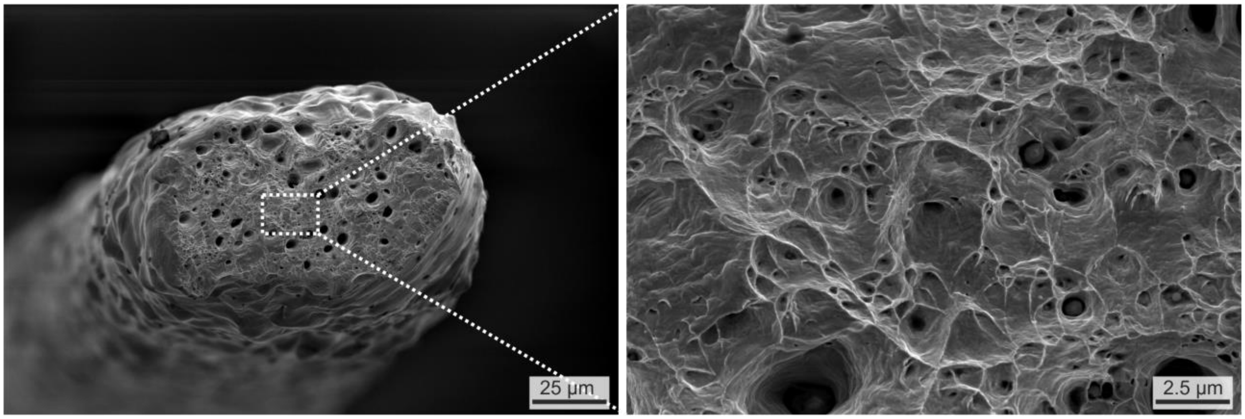

Supplementary Figure 6 Ductile fracture of 3D ink-extruded, reduced and sintered CoCrFeNi filament. Scanning electron micrographs of the fracture surface after tensile fracture of a CoCrFeNi filament illustrating that void coalescence is the dominant ductile fracture mechanism. left) Filament fracture surface, showing local necking and void formation. right) detailed view of the fracture surface, showing dimples typical of ductile failure.

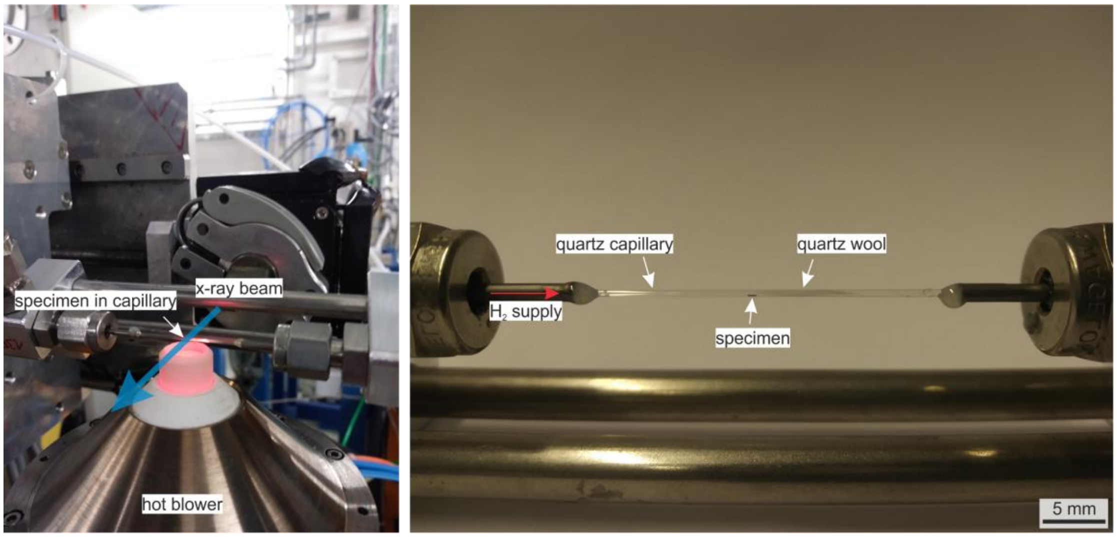

Supplementary Figure 7 Setup for in situ X-ray diffraction in H_2 at elevated temperature left) Arrangement of the specimen jig holding the quartz capillary with the printed filament specimen, horizontally-incoming x-ray beam (blue arrow) and the hot blower mounted vertically. Right) Detailed view of the 0.5 mm OD diameter quartz capillary, connected to the steel tubes fixed in Swagelok fittings, the co-reduced and sintered specimen in the center and a loose plug of quartz wool to keep it in place upon shrinkage. H_2 is flowed through the capillary and vented.

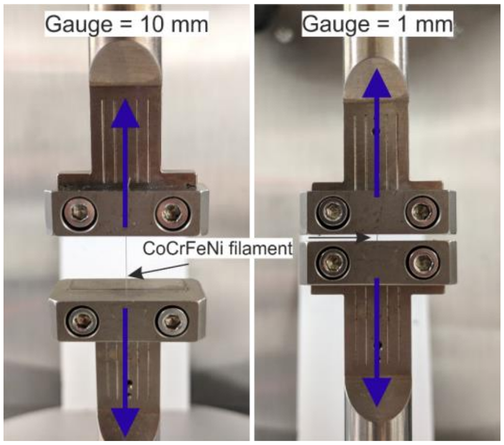

Supplementary Figure 8 Setup for tensile testing of single HEA filaments. HEA filaments in the RSA-G2 mechanical analyzer, prior to tensile testing, with a gauge length of 10 mm (left) and 1 mm (right). The filament is clamped directly (without tabs) with a torque of 0.50 Nm, and it is aligned centered in the load path (blue arrows).

Supplementary References

1. Huo, W. *et al.* Remarkable strength of CoCrFeNi high-entropy alloy wires at cryogenic and elevated temperatures. *Scr. Mater.* **141**, 125–128 (2017).
2. Huo, W. *et al.* Strain-rate effect upon the tensile behavior of CoCrFeNi high-entropy alloys. *Mater. Sci. Eng. A* **689**, 366–369 (2017).
3. Huang, T. *et al.* Effect of carbon addition on the microstructure and mechanical properties of CoCrFeNi high entropy alloy. *Sci. China Technol. Sci.* **61**, 117–123 (2018).
