# Supplementary material for: 3D ink-extrusion additive manufacturing of CoCrFeNi high-entropy alloy micro-lattices
Source: Nat Commun. 2019 Feb 22;10:904. doi: 10.1038/s41467-019-08763-4 (PMC6385271; doi:10.1038/s41467-019-08763-4)
Supplement: Supplementary file 3 — Description of Additional Supplementary Files [file 41467_2019_8763_MOESM3_ESM.pdf]

**Description of Additional Supplementary Files**

File Name: Supplementary Movie 1

Description: Compression testing of 3D ink-extruded and sintered CoCrFeNi HEA micro-lattices at room temperature showing extensive compressive deformation without failure. Frames were taken at 10% strain intervals.
