# Supplementary figures and images for: 3D ink-extrusion additive manufacturing of CoCrFeNi high-entropy alloy micro-lattices
Source: Nat Commun. 2019 Feb 22;10:904. doi: 10.1038/s41467-019-08763-4 (PMC6385271; doi:10.1038/s41467-019-08763-4)

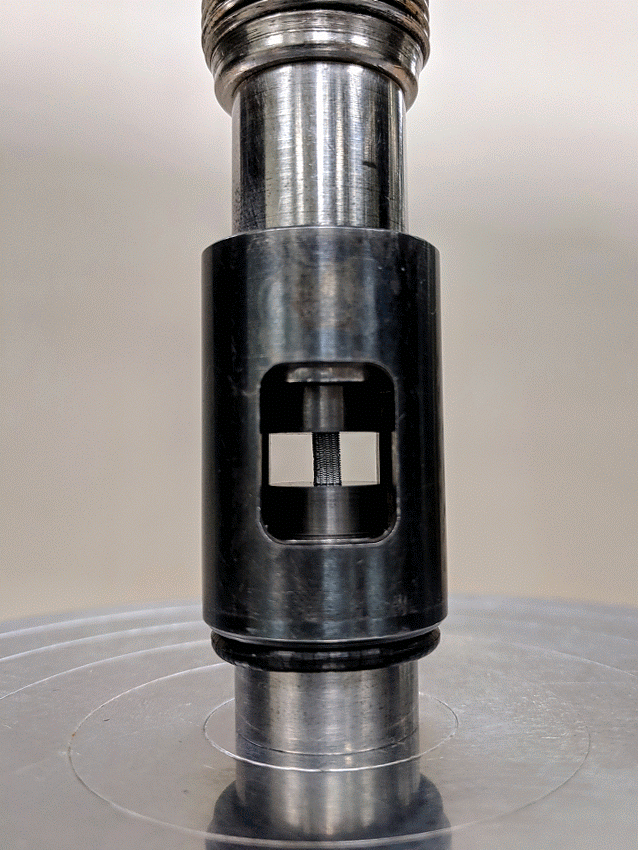

Supplement: Supplementary file 4 — Supplementary Movie 1 [file 41467_2019_8763_MOESM4_ESM.gif]
